# Circulating Saturated Fatty Acids and Incident Type 2 Diabetes: A Systematic Review and Meta-Analysis

**DOI:** 10.3390/nu11050998

**Published:** 2019-05-01

**Authors:** Lihua Huang, Jie-sheng Lin, Izzuddin M Aris, Guiyou Yang, Wei-Qing Chen, Ling-Jun Li

**Affiliations:** 1 Department of Medical Statistics and Epidemiology, Guangzhou Key Laboratory of Environmental Pollution and Health Assessment, Guangdong Provincial Key Laboratory of Food, Nutrition and Health, School of Public Health, Sun Yat-sen University, Guangzhou 510080, China; hlihua2@mail2.sysu.edu.cn (L.H.); linjsh6@mail2.sysu.edu.cn (J.-s.L.); yanggy7@mail2.sysu.edu.cn (G.Y.); 2 Division of Chronic Disease Research Across the Lifecourse, Department of Population Medicine, Harvard Medical School and Harvard Pilgrim Health Care Institute, Boston, MA 02215, USA; Izzuddin_Aris@sics.a-star.edu.sg; 3 Department of Obstetrics and Gynecology, Yong Loo Lin School of Medicine, National University of Singapore, Singapore 119074, Singapore; 4 Singapore Institute for Clinical Sciences, Agency for Science, Technology and Research, Singapore 117609, Singapore; 5 Department of Information Management, Xinhua College, Sun Yat-sen University, Guangzhou 510520, China; 6 Division of Obstetrics and Gynecology, KK Women’s and Children’s Hospital, Singapore 229899, Singapore; Queenie.Li.L@kkh.com.sg; 7 Obstetrics and Gynecology Academic Clinical Program, Duke-NUS Medical School, Singapore 169857, Singapore; 8 Singapore Eye Research Institute, Singapore National Eye Centre, Singapore 169856, Singapore

**Keywords:** type 2 diabetes, incidence, prospective cohort study, saturated fatty acids, circulating, meta-analysis, systematic review

## Abstract

The effect of saturated fatty acids (SFAs) on incident type 2 diabetes (T2D) is controversial and few have systematically appraised the evidence. We conducted a comprehensive search of prospective studies examining these relationships that were published in PubMed, Web of Science, or EMBASE from 21 February 1989 to 21 February 2019. A total of 19 studies were included for systematic review and 10 for meta-analysis. We estimated the summarized relative risk (RR) and 95% confidence interval (95% CI) using a random (if I^2^ > 50%) or a fixed effects model (if I^2^ ≤ 50%). Although the included studies reported inconclusive results, the majority supported a protective effect of odd-chain and an adverse impact of even-chain SFAs. Meta-analysis showed that the per standard deviation (SD) increase in odd-chain SFAs was associated with a reduced risk of incident T2D (C15:0: 0.86, 0.76–0.98; C17:0: 0.76, 0.59–0.97), while a per SD increase in one even-chain SFA was associated with an increased risk of incident T2D (C14:0: 1.13, 1.09–1.18). No associations were found between other SFAs and incident T2D. In conclusion, our findings suggest an overall protective effect of odd-chain SFAs and the inconclusive impact of even- and very-long-chain SFAs on incident T2D.

## 1. Introduction

Type 2 diabetes (T2D) is a complex metabolic disorder characterized by chronic hyperglycemia, mainly due to insulin resistance and/or abnormal insulin secretion [1]. T2D accounts for over 90% of all diabetic cases and was estimated to affect 425 million people worldwide aged from 20 to 79 years in 2017, with a projected increase to 629 million in 2045 [2]. T2D is also known to cause long term complications that lead to great healthcare burdens such as end-stage renal disease and non-traumatic lower extremity amputation [3,4]. In addition to the established traditional risk factors that are related to T2D (i.e., obesity, smoking status, and sedentary behaviors) [5,6], the role of high fat diet as a key contributor to T2D development has been increasingly recognized over the past few decades [7]. 

Fatty acids include unsaturated fatty acids (USFAs) and saturated fatty acids (SFAs). USFA intake has been widely accepted as beneficial for cardio-metabolic health, while SFAs have received very inconclusive opinions [8]. Since traditional research has suggested that total SFAs intake is associated with impaired insulin sensitivity, glucose intolerance, and T2D [9,10,11] possibly via its lipotoxicity [12,13,14], existing dietary guidelines have recommended that total SFA intake should not exceed 10% of the daily total energy intake [15]. However, new evidence has shown that there is an inverse cross-sectional association between SFA-rich dairy products and T2D [16]. Furthermore, a subsequent meta-analysis found that total SFAs was not associated with T2D, cardiovascular disease (CVD), and all-cause mortality [17]. Therefore, such findings have challenged the traditional belief that SFAs only lead to adverse health outcomes. 

To overcome these inconsistent findings, researchers have examined circulating SFAs individually, which could more accurately reflect its concentrations from both dietary intake and endogenous synthesis when compared to those collected from self-recalled reports [18]. A growing number of cohort studies have explored a wide spectrum of circulating SFAs, and reported equivocal associations between different chain lengths and incident T2D [19,20,21,22,23,24,25,26,27,28,29,30,31,32,33,34,35,36,37]. A recent pooled analysis based on 16 prospective cohorts in Caucasian populations (including unpublished data) showed that higher levels of odd-chain SFAs (C15:0 and C17:0) were associated with a lower risk of T2D [38]. However, since there might be significant cultural and genetic differences in dietary intake between Caucasians and other racial/ethnic groups, a genetic and geographic variation would be expected in the relationship between SFAs and T2D development. 

In order to address the gaps above-mentioned, we conducted a systematic review and meta-analysis on a wide spectrum of SFAs (i.e., odd-chain, even-chain, and very-long-chain) across different racial/ethnic groups. Based on the available evidence, we hypothesized that odd-chain SFAs were inversely associated with incident T2D, while other SFAs with different chain lengths had no significant associations on T2D development.

## 2. Materials and Methods

We performed the meta-analysis according to the Meta-analysis of Observation studies in Epidemiology (MOOSE) guidelines [39] and registered it in the International Prospective Register of Systematic Reviews (PROSPERO) with the registration number CRD42018110054. 

### 2.1. Literature Search

We searched papers from three online medical databases, namely, PubMed, Web of Science, and EMBASE that were published from 21 February 1989 to 21 February 2019. We used the keywords below for database searching: (i) “saturated fatty acid” or “saturated fatty acids”; (ii) “C14:0” or “tetradecanoic acid” or “myristic acid” or “C15:0” or “pentadecanoic acid” or “C16:0” or “hexadecanoic acid” or “palmitic acid” or “aethylic acid” or “C17:0” or “heptadecanoic acid” or “margaric acid” or “apurinic acid” or “C18:0” or “octadecanoic acid” or “stearic acid” or “C19:0” or “nonadecanoic acid” or “C20:0” or “eicosanoic acid” or “arachidic acid” or “docosanoic acid” or “arachidic acid” or “C21:0“ or “heneicosenoic acid” or “C22:0” or “docosanoic acid” or “behenic acid” or “C23:0” or “tricosanoic acid” or “C24:0” or “tetracosanoic acid” or “lignoceric acid” or “long-chain” or “very-long-chain” or “long chain” or “very long chain” or “odd-chain” or “even-chain” or “odd chain” or “even chain”; (iii) “circulating” or “serum” or “plasma” or “red blood cell” or “erythrocyte” or “blood”; (iv) “type 2 diabetes” or “T2D or diabetes mellitus” or “diabetes”; (v) “cohort” or “nested” or “prospective”. We performed the search by combining (i) or (ii) or (iii) and (iv) and (v). The initial search identified 1165 publications on 21 February 2019. 

### 2.2. Study Selection Criteria

**Figure 1:**
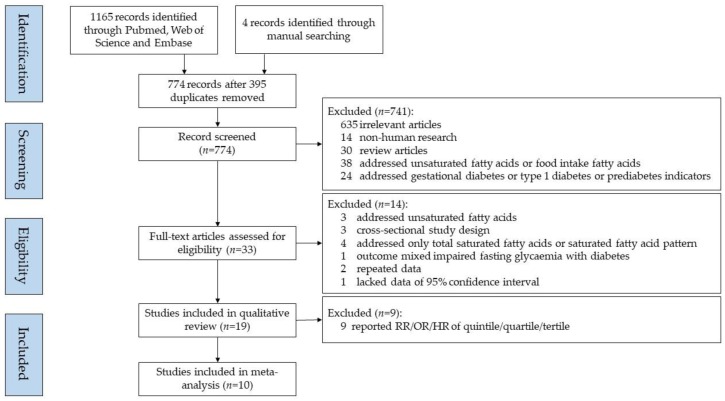
Flow diagram of the study selection.

One investigator (*L.H.*) determined the eligible studies by reviewing the titles and abstracts, and another investigator (*J.-s.L.*) verified the papers independently. The inclusion criteria of our analyses are listed below: (i) prospective study design on associations between SFAs and T2D; (ii) full-text available; (iii) written in English; (iv) reported odds ratios (OR), hazard ratios (HR), relative risk (RR) with 95% confidence intervals (CI) provided; (v) detailed description of SFAs assessment; (vi) original articles; (vii) published by 21 February 2019. Exclusion criteria included: (i) articles not written in English; (ii) case reports or reviews; (iii) animal studies; (iv) in vitro or in vivo studies; (v) articles that did not report the effect estimates (i.e., OR, HR, RR, or 95% CI). After the strict inclusion and exclusion screening, we selected 15 studies for systematic review. We additionally reviewed all of the included papers’ references for other potential eligible studies, of which four were further included in the systematic review. At the end of the search, we chose a total of 19 papers for systematic review. We only included 10 studies for meta-analysis that reported an OR/HR/RR with a 95% CI in incident T2D per standard deviation (SD) increase in SFAs. Figure 1 illustrates the flow diagram of the search strategy and study selection.

### 2.3. Data Extraction

One investigator (*L.H.*) conducted the data extraction and the two other investigators (*J.-s.L.* and *G.Y.*) verified the results independently. The extracted information included the first author’s last name, year of the publication, country or region of study location, study name, study samples, person-years of follow up or follow-up years, T2D assessment or definition or diagnosis, characteristics of the included participants (number of participants, age range at recruitment, and sex proportion), assessment of SFAs, variates adjusted for in the multivariable analysis as well as the adjusted RR, OR, HR with a 95% CI. 

### 2.4. Quality Assessments

One investigator (*L.H.*) performed the quality assessments for all papers based on the Newcastle–Ottawa Scale Criteria (NOSC) [40], and two other investigators (*J.-s.L.* and *G.Y.*) verified the findings independently. The maximum score of nine points in the Newcastle–Ottawa Scale are distributed in three aspects: (i) Selection of study groups up to four points (one point if each of the following is fulfilled: representativeness of the exposed cohort, selection of non-exposed cohort, ascertainment of exposure, and demonstration that outcome of interest was not present at the start of study); (ii) Comparability of groups up to two points (one point if each of the following is fulfilled: comparability of cohorts on the basis of the design and analysis controlled for confounders); and (iii) Assessment of exposure and outcomes up to three points (one point if each of the following is fulfilled: assessment of outcome, a minimum follow-up of 6 years, and at least 80% follow-up rate. We used the points to further categorize the publication quality as high (between 8–9 points), moderate (between 4–7 points), and low (between 0–3 points) [41]. 

### 2.5. Data Analysis

We used the Q and I^2^ statistic to assess heterogeneity across the studies [42]. For individual SFAs with three or more studies available, we calculated the summarized RR with a 95% CI in incident T2D per SD increase in SFAs by using either the fixed (if I^2^ <= 50%) or random effects model (if I^2^ > 50%) [43]. We reported the between-study heterogeneity in each meta-analysis performed and defined substantial/high and moderate heterogeneity as an I^2^ value greater than 75 and 50, respectively. To evaluate publication bias, we performed Egger’s test (linear regression method) and Begg’s test (rank correlation method), and a *p*-value <0.10 was considered representative of statistically significant publication bias [44,45]. We performed all statistical analyses using STATA version 12.0 (Stata Corporation, College Station, TX, USA) and defined statistical significance of two-tailed *p*-values as <0.05, unless otherwise specified.

## 3. Results

Table 1 and Table S1 summarize the characteristics and quality scores of the 19 studies included in our systematic review. All studies were conducted in North America (USA, *n* = 7) [21,24,28,30,31,32,34], Europe (*n* = 6) [20,22,23,27,29,33], Oceania (Australia, *n* = 2) [19,26], and Asia (Japan, *n* = 1, [35]; Singapore, *n* = 1, [37]; China, *n* = 2, [25,36]). Most studies (*n* = 17) included both sexes as study subjects, while two focused only on either women (one study in America) [32], or men (one study in Europe) [29]. Except for one study in China [25] that did not report the exact sample size, the remaining studies (*n* = 18) involved 63,050 participants (range: 187‒27,296) with a median follow-up period of seven years (range: 4‒15.2 years). In terms of SFA assessments, studies varied in their sample processing methods. For example, nine studies used plasma phospholipids [19,21,24,27,29,30,31,34], four used erythrocyte membranes fraction [20,23,25,32], four used serum lipids [28,33,35,37], one used whole blood sample [26], and one used both plasma phospholipids and erythrocyte membranes fraction [22]. Four studies identified T2D using self-reported information only [19,26,32,34], while the majority of studies (*n* = 12) diagnosed T2D using blood indicators according to the 1999 World Health Organization Guidelines or the 2014 American Diabetes Association Criteria (fasting plasma glucose value ≥7.0 mmol/L and/or non-fasting or 2-h glucose ≥11.1 mmol/L and/or glycated hemoglobin (HbAlc) ≥6.5%) [1,46]. Most studies adjusted for a wide range of potential confounders including age (*n* = 16), sex (*n* = 14), body mass index (BMI) (*n* = 15), physical activity (*n* = 18), alcohol intake (*n* = 17), smoking status (*n* = 17), and total energy intake (*n* = 9).

Table 2 summarizes the findings of each study included in the systematic review. Five out of 12 studies reported an overall protective effect of odd-chain SFAs on incident T2D, while other studies reported non-significant associations. Eight studies showed that at least one even-chain SFA was associated with an increased risk of incident T2D, while eight other studies did not find any significant associations. Nine studies investigated the association between very-long-chain SFAs with T2D. Three reported a protective relationship of at least one very-long-even-chain SFA with incident T2D, three studies showed no associations, and the remaining three studies reported associations with increased risk of incident T2D. Additionally, only one study investigated the association between heneicosanoic acid (C21:0) and incident T2D, however, no association was reported. One out of two studies showed that tricosanoic acid (C23:0) was inversely associated with incident T2D and another suggested a non-significant association. 

**Figure 2:**
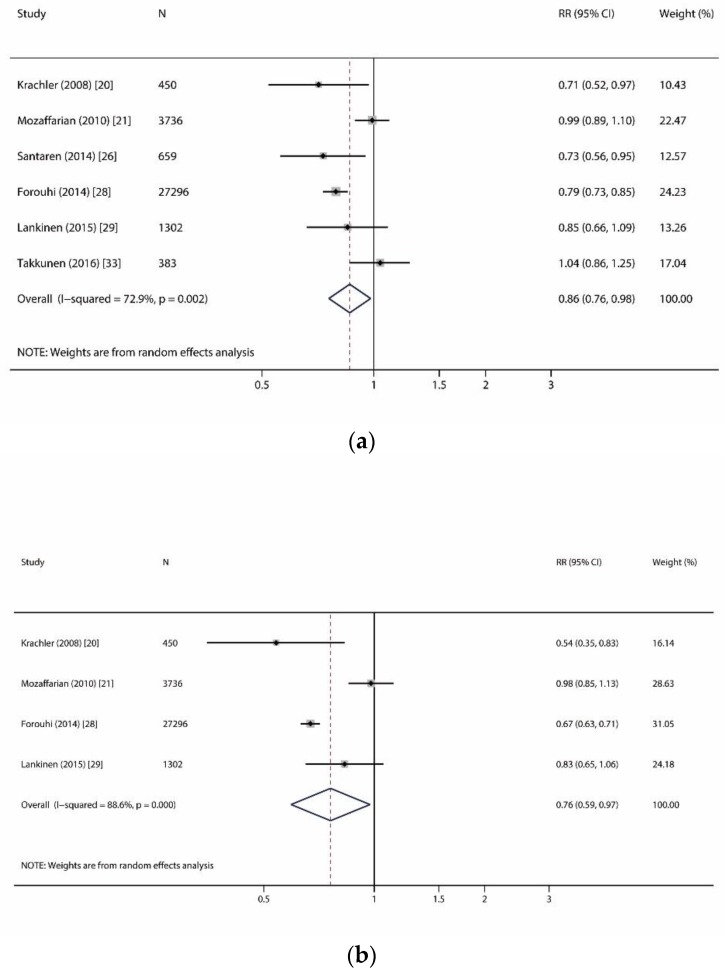
Forest plots of studies investigating the relationship of odd-chain saturated fatty acids and incident type 2 diabetes. (**a**) Forest plot for pentadecanoic acid (C15:0); (**b**) Forest plot for heptadecanoic acid (C17:0). RR: relative risk; CI: confidence interval.

**Figure 3:**
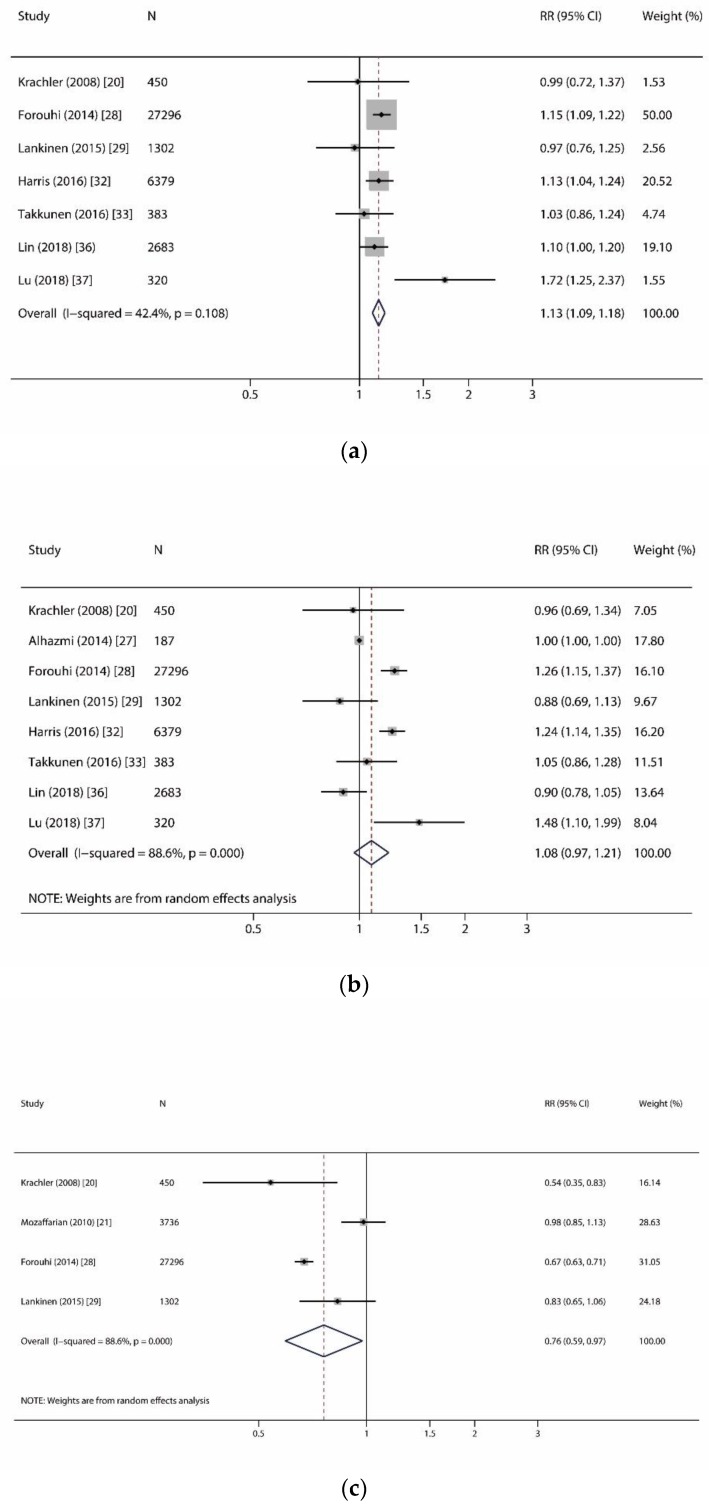
Forest plots of studies investigating the relationship of even-chain saturated fatty acids and incident type 2 diabetes. (**a**) Forest plot for myristic acid (C14:0); (**b**) Forest plot for palmitic acid (C16:0); (**c**) Forest plot for stearic acid (C18:0). RR: relative risk; CI: confidence interval.

**Figure 4:**
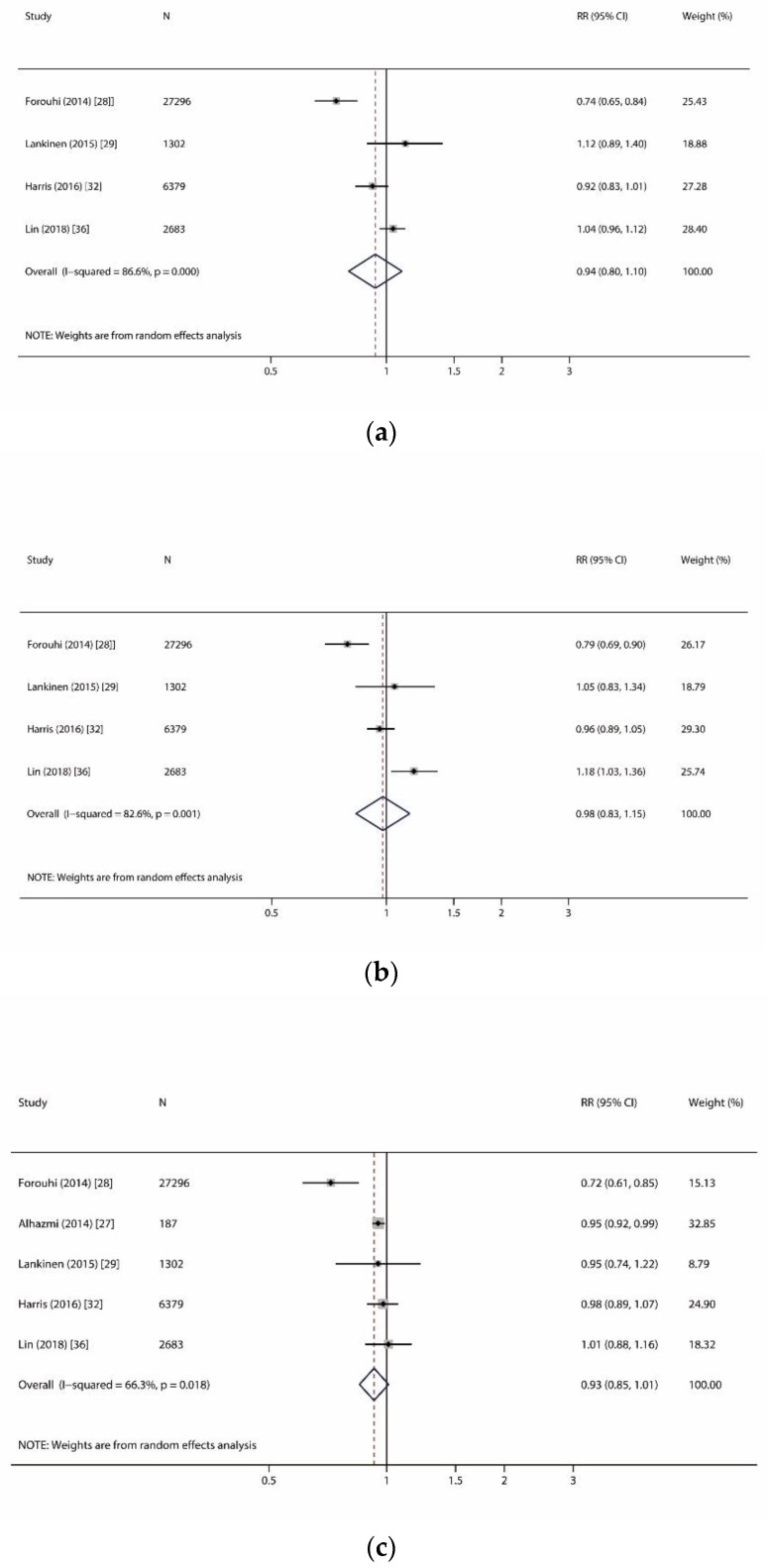
Forest plots of studies investigating the relationship of very-long-chain saturated fatty acids and incident type 2 diabetes. (**a**) Forest plot for arachidic acid (C20:0); (**b**) Forest plot for behenic acid (C22:0); (**c**) Forest plot for lignoceric acid (C24:0). RR: relative risk; CI: confidence interval.

Table S2 summarizes the characteristics and quality scores of the 10 studies included for the meta-analysis. All studies were defined as moderate-high quality (scored ≥5 points), which guaranteed a decent quality of our meta-analysis. Table 3 and Figure 2, Figure 3 and Figure 4 show the summarized RR of different SFAs (per 1 SD increase) on incident T2D. Figure 2 shows the meta-analysis on significant associations of odd-chain SFAs and reduced incident T2D. In Figure 2a, we conducted an analysis from six studies (12,924 T2D cases out of 33,826 participants) on the association between pentadecanoic acid (C15:0) and incident T2D [20,21,27,28,29,33]. For the per SD increase in C15:0, the summarized RR was 0.86 (95% CI: 0.76–0.98) with a moderate heterogeneity (I^2^ = 72.9%, *p* = 0.002). In Figure 2b, we analyzed heptadecanoic acid (C17:0) and incident T2D risk from four studies (12,666 T2D cases out of 32,784 participants) [20,21,27,29]. For the per SD increase in C17:0, the summarized RR was 0.76 (0.59–0.97) with substantial heterogeneity (I^2^ = 88.6%, *p* < 0.001).

We reported a significant association of myristic acid (C14:0) and incident T2D, but no association of other even-chain SFAs and incident T2D (Figure 3). In Figure 3a, we analyzed seven studies (13,596 T2D cases out of 38,813 participants) on the relationship of C14:0 and incident T2D [20,27,29,32,33,36,37]. For the per SD increase in C14:0, the summarized RR was 1.13 (1.09–1.18) with lower heterogeneity (I^2^ = 42.4%, *p* = 0.11). In Figure 3b,c, we investigated the associations of palmitic acid (C16:0) and stearic acid (C18:0) with incident T2D risk in eight studies (13,633 T2D cases out of 39,000 participants) [20,26,27,29,32,33,36,37]. For the per SD increase in C16:0 and C18:0, the summarized RR was 1.08 (0.97–1.21) with substantial heterogeneity (I^2^ = 88.6%, *p* < 0.001) and 1.05 (0.99–1.12) with moderate heterogeneity (I^2^ = 63.8%, *p* = 0.007), respectively. 

Similarly, we found no association and moderate to high heterogeneity (I^2^: 66.3–96.6%, all *p* < 0.01) between any very-long-even-chain SFAs (i.e., arachidic acid (C20:0), behenic acid (C22:0), and lignoceric acid (C24:0) and incident T2D (Figure 4). Given the limited number of studies performed on very-long-odd-chain SFAs, we were not able to conduct meta-analysis for C21:0 and C23:0. 

Table 3 shows the publication bias in our meta-analysis using Egger’s test and Begg’s test. Publication bias was only found on studies reporting an association of C18:0 and incident T2D (Egger’s test, *p* = 0.068 while Begg’s test, *p* = 0.174).

## 4. Discussion

Our systematic review and meta-analysis confirmed the protective effect of odd-chain SFAs (C15:0 and C17:0) and an adverse effect of only one even-chain SFAs (C14:0) on incident T2D, while no associations were observed between other even-chain SFAs (C16:0, C18:0, C20:0, C22:0, and C24:0) and incident T2D. 

Several studies have reported that high concentrations of odd-chain SFAs (C15:0 and C17:0) were correlated with a decreased risk of incident T2D [20,27]. Our meta-analysis on 10 studies supported this finding and was consistent with results from a pooled analysis on 16 prospective cohorts and a meta-analysis on 12 case-control studies [38,47]. In addition to being an energy source, fatty acids also play an important role in signaling metabolic regulations including gene expression, inflammatory and metabolic responses, and growth and survival pathways [48]. For example, Santaren et al. suggested that serum C15:0 was inversely associated with plasminogen activator inhibitor-1 (PAI-1), tumor necrosis factor-α (TNF-α), and interleukin-18 (IL-18) [49]. Another study by Zheng et al. also reported inverse associations between higher levels of odd-chain SFAs and lower levels of major lipids (i.e., total cholesterol, triglycerides, apolipoprotein A-1, apolipoprotein B) and hepatic markers [50]. However, we cannot rule out the likelihood that these observations may be confounded by other dietary components and various lifestyle habits. These factors should be considered in further studies. Given the possible protective role of circulating odd-chain SFAs on the risk of future diabetes, identifying the factors that affect their circulating concentrations may be of importance. The origin of odd-chain SFAs has long been attributed to diet, especially dairy product intake [51]. However, emerging evidence suggests that circulating C15:0 and C17:0 are independently derived. For instance, C15:0 correlates directly with dietary intake, while C17:0 is a product of biosynthesis regulated by dietary intake [52,53]. Interestingly, several epidemiological studies have shown that C17:0 has a stronger inverse association with metabolic diseases than C15:0 [27,54,55], which is consistent with our meta-analysis that showed more protective effect in C17:0 than C15:0 in relation to incident T2D. While verifying the results in our study, we also acknowledge that the heterogeneity of pooling SFAs data from different lipid fractions such as erythrocyte membranes fraction and plasma phospholipids may be significant. While we interpreted our findings carefully, we strongly recommend that further studies regarding the biosynthesis of C17:0 and how these pathways relate to T2D are very much needed. 

In terms of circulating even-chain SFAs (C14:0, C16:0, and C18:0), half of the previous studies reported an adverse effect on incident T2D. Furthermore, evidence showed that even-chain SFAs were positively associated with metabolic markers of the lipid, hepatic, glycemic, and inflammation pathways [49,50]. Unlike odd-chain SFAs, even-chain SFAs can be derived from both exogenous intake (i.e., typical Western diets rich in butter, palm oil, and red meat) and endogenous synthesis (i.e., de novo lipogenesis (DNL) pathway) [56,57]. The DNL pathway mainly synthesizes C16:0 and C18:0, while C14:0 seemed to be a minor product of this pathway [58]. Since dietary components (e.g., carbohydrate and alcohol intake), even at usual ranges of population exposures, were positively associated with circulating concentrations of fatty acids in the DNL pathway, the relationship of incident T2D and circulating even-chain SFAs (especially C16:0 and C18:0) might be affected by carbohydrate and alcohol intake [59]. However, our summarized results support that only C14:0 increased the risk of developing T2D, but not C16:0 and C18:0. One possibility might be due to the fact that many of our included studies did not adjust for either total energy intake or carbohydrate intake in their models, which might lead to the inconsistent findings among all even-chain SFAs. The other possibility might be due to the interaction between different SFAs entities. Existing studies suggest that different types of fatty acids are highly correlated [18,60,61,62,63], thus, the equivocal effect of even-chain SFAs on T2D development might be masked by different level of odd-chain SFAs and/or USFAs. Further studies investigating such associations should not only account for the fatty acid constitution, but also investigate the interaction (i.e., ratio) between even-chain and other forms of SFAs correlated with T2D incidence. 

Unlike odd-chain and even-chain SFAs, the relationship between very-long-chain SFAs and incident T2D has been understudied. Some research has shown that very-long-even-chain SFAs (C20:0, C22:0, and C24:0) are derived from limited food sources (i.e., peanuts, macadamia nuts, and canola oil) [64] and have been reported to be inversely associated with T2D development [65]. In contrast, very-long-even-chain SFAs have been suggested as the major backbone component of ceramide, which is known to be associated with increased insulin resistance and reduced β-cell mass and function [66,67,68]. Due to the contradicting mechanistic results regarding very-long-even-chain SFAs, studying their impact on T2D development will provide important insightful understanding of the metabolic pathophysiology in this form of SFA. Unfortunately, our systemic review showed contradictory associations between very-long-even-chain SFAs and incident T2D [26,27,29,32,36] and our meta-analysis did not find any associations between very-long-even-chain SFAs and incident T2D. Further studies in this group of SFAs, especially very-long-odd-chain SFAs, are needed. 

Interestingly, recent studies have shown that different forms of fatty acids may have synergistic effects on the development of T2D. Two studies have addressed the combined effect of fatty acids with T2D [69,70]; Kroger et al. showed that a high lipophilic index (the sum of individual fatty acid proportion multiplied with its respective melting point) was associated with a higher risk of T2D [69]. Additionally, Imamura et al. suggested that a combination of circulating fatty acids characterized by high concentrations of linoleic acid, odd-chain SFAs, and very-long-chain SFAs was associated with a lower incidence of T2D among high income Western populations [70]. The evidence on the interacting effect or patterns of these SFAs on the development of T2D, however, is still lacking. Future studies should focus on this in order to better understand the etiology of T2D led by SFAs.

The novelty of our review is that we systematically consolidated the evidence of the association between individual circulating SFAs and incident T2D based on prospective cohorts of good research quality. However, our results are not without limitations. First, all individual SFAs were measured at one time-point and intra-individual variation over time is possible. Second, between-study heterogeneity was moderate to high across all of the included studies. This might be mainly due to the different assessments of SFAs, diverse population, and dietary pattern, or favor as well as various major food sources of SFAs. Third, a meta-analysis is unable to address issues related to residual confounding. Even though most studies in this meta-analysis had been adjusted for age, physical activity, alcohol intake, smoking status, and family history of diabetes, total energy intake and carbohydrate intake were not included for adjustment in most of the studies. Fourth, our results were likely to have some misclassified outcomes since the diagnosis of T2D was not standardized and two studies used self-reported T2D due to pragmatic reasons. Last but not least, even though we covered all published data, our findings might be not generalizable to Black, Hispanic, or Asian populations due to limited data reported in these populations. 

## 5. Conclusions

In summary, this systematic review and meta-analysis of all published prospective cohorts suggest an overall protective effect of odd-chain SFAs (C15:0 and C17:0) and an adverse effect of even-chain SFAs (C14:0) in the development of T2D. However, our evidence did not show the association of any other even-chain SFAs (C16:0, C18:0, C20:0, C22:0, and C24:0) with incident T2D. Due to the high study heterogeneity attributed to a large variation in the assessments of circulating SFAs, future well-designed studies with a larger sample size, standardized assessments of SFAs, and longer follow-up are warranted to verify our findings and explore the synergistic or additive effects of fatty acids on incident T2D. Additional clinical and animal studies are also needed to better understand the underlying mechanisms in individual SFAs attributed to the development of T2D. 

## Figures and Tables

**Table 1:** Characteristics of the included studies for systematic review.

Author (Year)	Study *, Location	Follow-up (Year)	Total N (n Cases)	Age (year), Male (%)	Ascertainment of Diabetes	Individual SFAs	Lipid Fraction	Exposure Categories	Adjustment	NOSC Score
Hodge (2007) [19]	MCCS, Australia	4	3737 (346)	36–72, 41	Self-reported	C15:0, C16:0, C18:0	PL	Quintile	Age, sex, country of birth, family history of diabetes, physical activity, alcohol intake, BMI and WHR.	7
Krachler (2008) [20]	VIP, Sweden	5.4	450 (159)	40–60, 49	HbA1c, OGTT	C14:0, C15:0, C16:0, C17:0, C18:0	EM	Continuous	Alcohol intake, BMI, HbA1c.	7
Mozaffarian (2010) [21]	CHS, America	10	3736 (304)	≥65, 42	OGTT, medication	C15:0, C17:0,	PL	Continuous	Age, gender, race, education, enrollment site, smoking, BMI, waist circumference, coronary heart disease, physical activity, alcohol use, and consumption of carbohydrate, protein, red meat, whole-fat dairy foods, low-fat dairy foods, and total energy.	9
Patel (2010) [22]	EPIC-norfolk, Europe	10	383 (199)	40–79, 47	Self-reported, medication	C14:0, C15:0, C16:0, C17:0, C18:0	PL/EM	Tertile	Age, sex, family history of diabetes, BMI, smoking status, physical activity, and alcohol intake.	9
Kröger (2011) [23]	EPIC-Potsdam, Europe	7	2724 (412)	35–65, 43	Self-reported, medication	C14:0, C15:0, C16:0, C17:0, C18:0, C20:0, C21:0, C22:0, C23:0, C24:0	EM	Quintile	Age, sex, BMI, waist circumference, cycling, sports activity, education, smoking status, alcohol intake, occupational activity, coffee intake and fiber intake.	9
Mozaffarian (2013) [24]	MESA, America	5	2281 (205)	45–84, 47	Fasting glucose, medication	C14:0, C15:0	PL	Quintile	Age, sex, race-ethnicity, education, field center, smoking status, alcohol use, physical activity, BMI, and waist circumference, dietary consumption of whole-fat dairy foods, low-fat dairy foods, red meat, and total energy.	8
Zong (2013) [25]	NHAPC, China	6	not available	50–70, 45	Fasting glucose, medication	C16:0	EM	Quartile	Age, sex, region, residence, physical activity, educational attainment, current smoking, BMI, current drinking, family history of diabetes, total energy intake, percentage of energy intake from carbohydrate, and energy-adjusted dietary GI.	8
Santaren (2014) [28]	IRAS, America	5	659 (103)	40–60, 45	OGTT	C15:0	SL	Continuous	Age, sex, ethnicity, center, physical activity, smoking status, alcohol intake, education, and total energy, fruit and vegetable, red meat, soft drink and fiber intakes.	8
Lemaitre (2015) [30]	CHS, America	10	3179 (284)	≥65, 39	Fasting glucose, non-fasting glucose and medication	C20:0, C22:0, C24:0	PL	Quartile	Age sex, race, clinic, education, smoking, alcohol use, BMI, waist circumference, physical activity, treated hypertension, prevalent ischemic heart disease, and self-reported health status at baseline.	9
Ma (2015) [31]	CHS, America	10	3004 (297)	≥65, 40	Fasting glucose, non-fasting or 2h-glucose and medication	C14:0, C16:0, C18:0	PL	Quintile	Age, sex, race, education, clinic, smoking status, alcohol consumption, leisure time physical activity, prevalence of ischemic heart disease, hypertension at baseline, BMI, protein, waist circumference, consumption of carbohydrate, and total energy.	9
Alhazmi (2014) [26]	HCS, Australia	5	187 (37)	55–85, 51	Self-reported	C16:0, C18:0, C24:0	WB	Continuous	Age and gender, BMI; physical activity; alcohol intake; smoking; supplement use, carbohydrate, fiber, and protein.	6
Forouhi (2014) [27]	EPIC-InterAct, Europe	11.7	27,296 (12,132)	53.7(mean), 38	Self-reported, care registers, hospital admissions, mortality data, medication	C14:0, C15:0, C16:0, C17:0, C18:0, C20:0, C22:0, C24:0	PL	Continuous	Age, sex, center, physical activity, smoking status, and education level, total energy intake, alcohol intake, and BMI.	9
Lankinen (2015) [29]	METSIM, Finland	5.9	1302 (71)	45–68, 100	OGTT, HbAlc	C14:0, C15:0, C16:0, C17:0, C18:0, C20:0, C22:0, C24:0	PL	Continuous	Age, BMI, smoking, physical activity and fasting glucose at baseline.	5
Harris (2016) [32]	WHIMS, America	11	6379 (703)	65–80, 0	Self-reported	C14:0, C16:0, C18:0, C20:0, C22:0, C24:0	EM	Continuous	Age, race, waist circumference, highest education, current smoking status, physical activity, weekly alcohol intake, glycemic load, and family history of diabetes.	7
Takkunen (2016) [33]	FDPS, Finland	11	383 (155)	40–65, 33	OGTT	C14:0, C15:0, C16:0, C18:0	SL	Continuous	Age, sex, study group, smoking, alcohol intake, waist circumference and physical activity at leisure time, study centers, fiber intake, carbohydrate intake, energy intake and serum triglyceride concentration, concentrations of plasma fasting and 2-h glucose.	8
Yakoob (2016) [34]	NHS and HPFUS, America	15.2	3333 (277)	30–75, 44	Self-reported	C14:0, C15:0, C17:0	PL	Quartile	Age, race, smoking status, physical activity, alcohol, family history of diabetes mellitus, parental history of MI, hypertension, hypercholesterolemia, menopausal status, postmenopausal hormone use, and consumption of fish, processed meats, unprocessed meats, fruits, vegetables, whole grains, coffee, sugar-sweetened beverages, glycemic load, dietary calcium, total energy, polyunsaturated fat, and plasma trans-18:1, trans-18:2, 16:0, and 18:0.	7
Akter (2017) [35]	Hitachi Health Study, Japan	5	1014 (336)	34–69, 91	HbA1c, fasting or non-fasting glucose, medication	C14:0, C15:0, C16:0, C17:0, C18:0, C20:0	SL	Quartile	Age, sex, and month of examination, leisure-time physical activity, occupational physical activity, smoking status, alcohol consumption, shift work, sleep duration, family history of diabetes, and hypertension, BMI.	7
Lin (2018) [36]	GNHS, China	5.6	2683 (216)	40–75, 33	Fasting glucose, HbAlc, medications	C14:0, C16:0, C18:0, C20:0, C22:0, C24:0	EM	Quintile/Continuous	Sex, BMI, WHR, smoking status, alcohol drinking, tea drinking, education level, household income, physical activity, family history of diabetes, total energy intake, low-density lipoprotein cholesterol, high-density lipoprotein cholesterol, triglycerides and fasting glucose.	8
Lu (2018) [37]	SCHS, Singapore	6	320 (160)	60–70, 49	HbAlc	C14:0, C16:0, C18:0	SL	Tertile/Continuous	BMI, history of hypertension, smoking, physical activity, fasting status, HDL-cholesterol, triglycerides, random glucose and HbA1c levels.	8

* Abbreviations: ARIC: the Atherosclerosis Risk in Communities Study; MCCS: Melbourne Collaborative Cohort Study; VIP: Vasterbotten Intervention Programme; EPIC: European Prospective Investigation into Cancer and nutrition; CHS: Cardiovascular Health Study; MESA: the Multi-Ethnic Study of Atherosclerosis; NHAPC: The Nutrition and Health of Aging Population in China study; IRAS: the Insulin Resistance Atherosclerosis Study; HCS: Hunter Community Study; METSIM: metabolic syndrome in men; FDPS: Finnish Diabetes Prevention Study; WHIMS: Women’s Health Initiative Memory Study; NHS: Nurses’ Health Study; HPFUS: Health Professionals Follow-Up Study; GNHS: Guangzhou Nutrition and Health Study; SCHS: Singapore Chinese Health School; PL: plasma phospholipid; EM: Erythrocyte membranes; SL: serum lipids; WB: whole blood; BMI: Body Mass Index; WHR: waist-to-hip ratio; MI: myocardial infarction; GI: glycemic index; HDL: high density lipoprotein; HbAlc: glycated hemoglobin; OGTT: oral glucose tolerance test; NOSC: Newcastle–Ottawa Scale Criteria.

**Table 2:** Study-specific results of individual SFAs and incident T2D.

Author (Year)	Myristic Acid (C14:0)	Pentadecanoic Acid (C15:0)	Palmitic Acid (C16:0)	Heptadecanoic Acid (C17:0)	Stearic Acid (C18:0)	Arachidic Acid (C20:0)	Heneicosanoic Acid (C21:0)	Behenic Acid (C22:0)	Tricosanoic Acid (C23:0)	Lignoceric Acid (C24:0)
***Per each SD increment***										
Krachler (2008) [20]	○	↓	○	↓	○					
Mozaffarian (2010) [21]		○		○						
Alhazmi (2014) [26]			○		○					↓
Forouhi (2014) [27]	↑	↓	↑	↓	↑	↓		↓	↓	↓
Santaren (2014) [28]		↓								
Lankinen (2015) [29]	○	○	○	○	↑	○		○		○
Harris (2016) [32]	↑		↑		○	○		○		○
Takkunen (2016) [33]	○	○	○		○					
Lin (2018) [36]	↑		○		○	○		↑		○
Lu (2018) [37]	↑		↑		↑					
***Highest vs. lowest***										
Hodge (2007) [19]		↓	○		↑					
Patel (2010) [22]	○	○	↑	↓	↓/○ *					
Kröger (2011) [23]	○	○	○	○	○	↓	○	○	○	↑
Mozaffarian (2013) [24]	○	○								
Zong (2013) [25]			↑							
Lemaitre (2015) [30]						↓		↓		↓
Ma (2015) [31]	○		↑		↑					
Yakoob (2016) [34]	○	↓		↓						
Akter (2017) [35]	○	○	○	○	○	○				
Lin (2018) [36]	○		↓		↑	↑		○		○
Lu (2018) [37]	↑		↑		↑					

Abbreviation: SFAs: saturated fatty acids; T2D: type 2 diabetes; SD: standard deviation. ↑: positive association; ○: no association; ↓: negative association; * For the Patel (2010) study, two kinds of lipid fraction (plasma phospholipid and erythrocyte-membrane phospholipid) were used for fatty acid measurement, and for C18:0, measurements in plasma phospholipid were negatively associated with incident T2D, but measurements in erythrocyte-membrane phospholipid was not associated with incident T2D.

**Table 3:** Main meta-analyses result of the relationship between individual SFAs and T2D (per SD difference).

Saturated Fatty Acids	No. of Studies	Total N (*n* Cases)	Follow-up Years (Mean) *	Summary Estimate (95% CI)	*P*	Heterogeneity Test	Effect Model	*P*_Begg_	*P*_Egger_
**Odd-chain SFAs**									
Pentadecanoic acid (C15:0)	6	33,826 (12,924)	11.1	0.86 (0.76, 0.98)	0.023	*p* = 0.002, I^2^ = 72.9%	R	0.707	0.950
Heptadecanoic acid (C17:0)	4	32,784 (12,666)	11.2	0.76 (0.59, 0.97)	0.030	*p* < 0.001, I^2^ = 88.6%	R	1.000	0.606
**Even-chain SFAs**									
Myristic acid (C14:0)	7	38,813 (13,596)	10.8	1.13 (1.09, 1.18)	<0.001	*p* = 0.108, I^2^ = 42.4%	F	0.368	0.863
Palmitic acid (C16:0)	8	39,000 (13,633)	10.8	1.08 (0.97, 1.21)	0.169	*p* < 0.001, I^2^ = 88.6%	R	0.902	0.199
Stearic acid (C18:0)	8	39,000 (13,633)	10.8	1.05 (0.99, 1.12)	0.119	*p* = 0.007, I^2^ = 63.8%	R	0.174	0.068
**Very-long-chain SFAs**									
Arachidic acid (C20:0)	4	37,660 (13,122)	10.9	0.94 (0.80, 1.10)	0.413	*p* < 0.001, I^2^ = 86.6%	R	0.734	0.773
Behenic acid (C22:0)	4	37,660 (13,122)	10.9	0.98 (0.83, 1.15)	0.792	*p* = 0.001, I^2^ = 82.6%	R	0.734	0.825
Lignoceric acid (C24:0)	5	37,847 (13,159)	10.9	0.93 (0.85, 1.01)	0.089	*p* = 0.018, I^2^ = 66.3%	R	0.806	0.627

Abbreviation: SFAs: saturated fatty acids; T2D: type 2 diabetes; SD: standard deviation; CI: confidence interval; R: random; F: fixed. * Follow-up years (mean) were calculated as the number of participants per study multiplied by the years of follow-up per study divided by the total number of participants.

## Supplementary Materials

The following are available online at https://www.mdpi.com/2072-6643/11/5/998/s1, Table S1: Quality score of included studies for systemic review and meta-analysis; Table S2: Characteristics of included studies for meta-analysis (Per 1 SD increase).
