# Supplementary material for: Circulating Saturated Fatty Acids and Incident Type 2 Diabetes: A Systematic Review and Meta-Analysis
Source: Nutrients. 2019 May 1;11(5):998. doi: 10.3390/nu11050998 (PMC6566227; doi:10.3390/nu11050998)
Supplement: Supplementary file 1 [file nutrients-11-00998-s001.pdf]

Table S1. Quality score of included studies for systemic review and meta-analysis.

Author (year)	NOS score	Selection				Comparability		Outcome		
		Representativeness of the exposed cohort	Selection of non-exposed cohort	Ascertainment of exposure	Demonstration that outcome of interest was not present at start of study	Study controls for the most important factor	Study controls for any additional factors	Assessment of outcome	Was follow-up long enough for outcomes to occur £	Adequacy of follow up of cohorts §
Hodge (2007)	7	*	*	*	*	*	*	/	/	*
Krachler (2008)	7	*	*	*	*	*	/	*	/	*
Patel (2010)	9	*	*	*	*	*	*	*	*	*
Mozaffarian (2010)	9	*	*	*	*	*	*	*	*	*
Kröger (2011)	9	*	*	*	*	*	*	*	*	*
Mozaffarian (2013)	8	*	*	*	*	*	*	*	/	*
Zong (2013)	8	*	*	*	*	*	*	*	*	/
Santaren (2014)	8	*	*	*	*	*	*	*	/	*
Alhazmi (2014)	6	*	*	*	*	*	*	/	/	/
Forouhi (2014)	9	*	*	*	*	*	*	*	*	*
Lankinen (2015)	5	/	*	*	*	*	/	*	/	/
Lemaitre (2015)	9	*	*	*	*	*	*	*	*	*
Ma (2015)	9	*	*	*	*	*	*	*	*	*
Takkunen (2016)	8	/	*	*	*	*	*	*	*	*
Harris (2016)	7	/	*	*	*	*	*	/	*	*
Yakoob (2016)	7	/	*	*	*	*	*	/	*	*
Akter (2017)	7	/	*	*	*	*	*	*	/	*
Lin (2018)	8	*	*	*	*	*	*	*	/	*
Lu (2018)	9	*	*	*	*	*	*	*	*	*

£ Star was given if the study had a minimum follow-up of six years.
§ Star was given if the study had a maximum loss rate of 20%.

Table S2. Characteristics of included studies for meta-analysis (Per 1 SD increase).

Author (year)	Study, location	Follow-up (year)	Total N (n cases)	Age (year), Male (%)	Ascertainment of Diabetes	Individual SFAs	Lipid fraction	Adjustment	NOSC score
Krachler (2008)	VIP, Sweden	5.4	450 (159)	40–60, 49	HbA1c, OGTT	C14:0, C15:0, C16:0, C17:0, C18:0	EM	Alcohol intake, BMI, HbA1c.	7
Mozaffarian (2010)	CHS, America	10	3736 (304)	≥65, 42	OGTT, medication	C15:0, C17:0,	PL	Age, gender, race, education, enrollment site, smoking, body mass index, waist circumference, coronary heart disease, physical activity, alcohol use, and consumption of carbohydrate, protein, red meat, whole-fat dairy foods, low-fat dairy foods, and total energy.	9
Santaren (2014)	IRAS, America	5	659 (103)	40–60, 45	OGTT	C15:0	SL	Age, sex, ethnicity, center, physical activity, smoking status, alcohol intake, education, and total energy, fruit and vegetable, red meat, soft drink and fiber intakes.	8
Forouhi (2014)	EPIC-InterAct, Europe	11.7	27,296 (12,132)	53.7(mean), 38	Self-reported, care registers, hospital admissions, mortality data, medication	C14:0, C15:0, C16:0, C17:0, C18:0, C20:0, C22:0, C24:0	PL	Age, center, sex, physical activity, smoking status, and education level, total energy intake, alcohol intake, and BMI.	9
Alhazmi (2014)	HCS, Australia	5	187 (37)	55–85, 51	Self-reported	C16:0, C18:0, C24:0	WB	Age and gender, BMI; physical activity; alcohol intake; smoking; supplement use, carbohydrate, fiber, and protein.	6
Lankinen (2015)	METSIM, Finland	5.9	1302 (71)	45–68, 100	OGTT, HbAlc	C14:0, C15:0, C16:0, C17:0, C18:0, C20:0, C22:0, C24:0	PL	Age, BMI, smoking, physical activity and fasting glucose at baseline.	5
Takkunen (2016)	FDPS, Finland	11	383 (155)	40–65, 33	OGTT	C14:0, C15:0, C16:0, C18:0	SL	Age, sex, study group, smoking, alcohol intake, waist circumference and physical activity at leisure time, study centers, fiber intake, carbohydrate intake, energy intake and serum triglyceride concentration, concentrations of plasma fasting and 2-h glucose.	8
Harris (2016)	WHIMS, America	11	6379 (703)	65–80, 0	Self-reported	C14:0, C16:0, C20:0, C22:0, C24:0	EM	Age, race, waist circumference, highest education, current smoking status, physical activity, weekly alcohol intake, glycemic load, and family history of diabetes.	7
Lin (2018)	GNHS, China	5.6	2683 (216)	40–75, 33	Fasting glucose, HbAlc, medications	C14:0, C16:0, C18:0, C20:0, C22:0, C24:0	EM	Sex, BMI, WHR, smoking status, alcohol drinking, tea drinking, education level, household income, physical activity, family history of diabetes, total energy intake, low-density lipoprotein cholesterol, high-density lipoprotein cholesterol, triglycerides and fasting glucose.	8
Lu (2018)	SCHS, Singapore	6	320 (160)	60–70, 49	HbAlc	C14:0, C16:0, C18:0	SL	BMI, history of hypertension, smoking, physical activity, fasting status, triglycerides, HDL-cholesterol, random glucose and HbA1c levels.	8

Abbreviations: VIP: Vasterbotten Intervention Programme; CHS: Cardiovascular Health Study; IRAS: The Insulin Resistance Atherosclerosis Study; EPIC: European Prospective Investigation into Cancer and nutrition; HCS: Hunter Community Study; METSIM: metabolic syndrome in men; FDPS: Finnish Diabetes Prevention Study; WHIMS: Women's Health Initiative Memory Study; GNHS:

Guangzhou Nutrition and Health Study; SCHS: Singapore Chinese Health School; L: plasma phospholipid; EM: Erythrocyte membranes; SL: serum lipids; WB: whole blood; BMI: Body Mass Index; WHR: waist-to-hip ratio; HDL: high density lipoprotein; HbAlc: glycated hemoglobin; OGTT: oral glucose tolerance test; NOSC: Newcastle–Ottawa Scale Criteria; SD: standard deviation.
